# An open presurgery MRI dataset of people with epilepsy and focal cortical dysplasia type II

**DOI:** 10.1038/s41597-023-02386-7

**Published:** 2023-07-20

**Authors:** Fabiane Schuch, Lennart Walger, Matthias Schmitz, Bastian David, Tobias Bauer, Antonia Harms, Laura Fischbach, Freya Schulte, Martin Schidlowski, Johannes Reiter, Felix Bitzer, Randi von Wrede, Atilla Rácz, Tobias Baumgartner, Valeri Borger, Matthias Schneider, Achim Flender, Albert Becker, Hartmut Vatter, Bernd Weber, Louisa Specht-Riemenschneider, Alexander Radbruch, Rainer Surges, Theodor Rüber

**Affiliations:** 1 Department of Epileptology, University Hospital Bonn, Bonn, Germany; 2 Department of Neurosurgery, University Hospital Bonn, Bonn, Germany; 3 Medical Faculty, University Hospital Bonn, Bonn, Germany; 4 Section of Translational Epilepsy Research, Department of Neuropathology, University Hospital Bonn, Bonn, Germany; 5 Institute of Experimental Epileptology and Cognition Research, University Hospital Bonn, Bonn, Germany; 6 Chair of Civil Law, Information Law and Data Law, Faculty of Law, University of Bonn, Bonn, Germany; 7 Department of Neuroradiology, University Hospital Bonn, Bonn, Germany

**Keywords:** Epilepsy, Pathology

## Abstract

Automated detection of lesions using artificial intelligence creates new standards in medical imaging. For people with epilepsy, automated detection of focal cortical dysplasias (FCDs) is widely used because subtle FCDs often escape conventional neuroradiological diagnosis. Accurate recognition of FCDs, however, is of outstanding importance for affected people, as surgical resection of the dysplastic cortex is associated with a high chance of postsurgical seizure freedom. Here, we make publicly available a dataset of 85 people affected by epilepsy due to FCD type II and 85 healthy control persons. We publish 3D-T1 and 3D-FLAIR, manually labeled regions of interest, and carefully selected clinical features. The open presurgery MRI dataset may be used to validate existing automated algorithms of FCD detection as well as to create new approaches. Most importantly, it will enable comparability of already existing approaches and support a more widespread use of automated lesion detection tools.

## Background & Summary

Focal cortical dysplasia (FCD) is a malformation of cortical development that frequently causes drug-resistant focal epilepsy. In drug-resistant individuals, epilepsy surgery often remains the most successful option for treatment. FCDs are the most commonly resected epileptogenic lesions in children^1^ and the third most common in adults^2^. Clinical manifestation of epilepsy due to FCDs usually occurs in early childhood, but later onset is not uncommon^3^. Seizure semiology depends on the location of FCD, which can occur in any brain lobe. The distribution across the cortical lobes is non-uniform, and in particular, FCD type II occurs most frequently in the frontal lobe^2,4,5^. FCDs were initially histopathologically categorized according to Palmini^6^. This system was first revised in 2011^7^ and most recently in 2022^8^ by the International League Against Epilepsy (ILAE). Several imaging features have been described. These are cortical thickness, blurring of the gray-matter/white-matter junction, gyration anomalies, and focally increased signals in the subcortical white matter, which sometimes extend to the ventricle as a so-called “transmantle sign”^9^. Nevertheless, FCDs often escape conventional neuroradiological diagnosis because these abnormalities can be very subtle. In such cases, finding the correct diagnosis can be challenging, and people with epilepsy must undergo extensive diagnostics (e.g., invasive EEG). So-called “MRI negative” cases of people with focal epilepsy but without visible MRI abnormalities have a lower chance of undergoing epilepsy surgery and show a significantly worse surgical outcome^10,11^. The success of epilepsy surgery depends on the accurate identification and complete excision of the epileptogenic zone. However, the chance of seizure freedom after epilepsy surgery is exceptionally high in people with epilepsy due to FCD^12^. Various automated or semi-automated postprocessing approaches have been developed in the past few years to optimize the visualization of FCDs^13–24^. David *et al*.^24^, used parts of this dataset (58 individuals) for the independent validation of an artificial neural network (ANN) for robust automated detection of FCDs based on morphometric feature maps generated by the Morphometric Analysis Program (MAP)^14,24^. Several other descriptive studies are also based on parts of this dataset^3,25–28^.

There are three major problems for FCD-detection-algorithms trained and validated using single-center datasets: One is that cohorts often are too small to develop a robust classification algorithm. Second, studies may recruit training and testing data from the same sample of people with epilepsy (internal validation), which is why their performance is overrated. Third, radiological diagnoses or ratings of MRI of individuals with FCD may strongly vary from site to site. The MRI volume of an individual with FCD may be described as “MR-negative” in one site and as “radiologically described FCD” in another site with corresponding effects on the evaluation of the respective detection algorithms. It, therefore, is of importance that the same well-annotated and sufficiently large dataset is being used for the validation of different algorithms and that this dataset has not been used for the training of these algorithms. Gill and colleagues overcame these problems by developing a multicenter-validated deep learning detection algorithm for FCD^15^. This algorithm has been trained and validated on a multicenter dataset with many people with histologically confirmed FCD. The authors note that the dataset contains information that could compromise the privacy of research participants, which is why the dataset is not publicly available^15^.

With the publication of the open presurgery MRI dataset of people with epilepsy due to focal cortical dysplasia and of control persons, we aim to overcome the abovementioned challenges. We hope to enable fellow researchers to measure the generalizability of automated detection methods, so-called external validation. Approaches are best validated and compared on such an external benchmark dataset. Additionally, this dataset allows the improvement of existing approaches by training them on more data. The longstanding goal of this publication is to optimize existing or new approaches for automated lesion detection until they become part of routine clinical practice and are not exclusively reserved for specialized clinics. Improved focus localization in imaging with a widely used tool may reduce the need for invasive diagnostics and the associated health risks and healthcare system costs.

## Methods

### Study approval

The ethics committee of the University of Bonn (Lfd.-Nr. 346/21) approved this study including anonymized publication of the MRI datasets as well as selected clinical/demographical characteristics of people with epilepsy and control persons. It adheres to the General Data Protection Regulation of the European Parliament and the Council. It has been conducted in close collaboration with the Data Protection Officer of the University of Bonn Medical Center (AF) and it includes only data of people who provided written and informed consent for the publication of their data as adults.

### Dataset selection

We first identified all people with epilepsy treated at the Department of Epileptology at the University Hospital Bonn from 2006 to 2021 due to histologically verified FCD type II or radiologically suspected FCD type II. We selected all who were over 18 years of age at the time of the conduction of our study. Individuals who were minor at the time the MRI conducted, were only included if they were adults at the time their data were prepared for publication. In parallel, we ascertained data from healthy control persons over 18 years of age. These people were contacted again and informed about the planned publication of anonymized MRI data in conjunction with selected demographical characteristics. People willing to participate in the study then received an information letter. We were able to contact 92 of 137 individuals with epilepsy and FCD by telephone. Of these, 85 (92.4%) gave informed consent for their MRI data and associated clinical characteristics to be made available anonymously to the scientific public. Accordingly, we included 85 control persons who consented to publication of their data in this study. Clinical characteristics of people with epilepsy were retrieved from the clinical records of the department of epileptology. The anatomical location of the FCD was indicated by two neuroradiologists. Complicated cases were discussed in a joint conference of experienced neuroradiologists and epileptologists, taking into account the results of all diagnostic modalities. Only in one case, the FCD could only be found after MAP^14^ was performed, which is why the initial MRI assessment by the neuroradiologist did not include it.

After the lesion location was determined, the definition of the region of interest (ROI) was conducted by the collaboration of two board-certified neurologists (see *Region of Interest* below). We summarized MRI diagnoses as “suspected FCD II”, “no abnormalities” ( = “MRI-negative”), and “other” (meaning that abnormalities were described as part of the clinical routine, but no FCD-suspect lesion was present; in these cases, the diagnosis was made only by histology). For histologically confirmed FCDs, we included only cases of FCD II (FCD IIa or IIb). FCD I or III were not included. Histological classification of FCDs was performed at the Department of Neuropathology of the University Hospital Bonn according to the ILAE classification^7^. For FCD type II, the results of the ILAE classification^7,8^ are identical to those of the Palmini classification^6^.

For those people who underwent epilepsy surgery, postoperative outcome was assessed 12 months after epilepsy surgery and at the latest clinical follow-up according to the Engel classification^29^. We also recorded the age at the MRI scan and the sex for all subjects. In people with epilepsy, age at first epileptic seizure was also ascertained.

Whether additional diagnostics (PET-CT, SPECT, MAP analysis^14^ or an invasive EEG) were performed in people with epilepsy can be found in the participants.tsv file^30^.

### Demographics and clinical characteristics of the published data

Of the 85 people with epilepsy who participated in the study, 35 (41.2%) are female and 50 (58.8%) are male. The mean age at onset of the first epileptic seizure was 10 years (min.: 0.5 years; max.: 41 years; median: 8 years). At the time of MRI Scan, the mean age was 28.9 years (min.: 12 years; max.: 65 years; median: 25 years), and the mean age at epilepsy surgery was 29.2 years (min.: 12; max.: 59 years; median: 27 years). The average age of the control persons at the time of the MRI scan was matched with the age of people with epilepsy (min.: 22 years, max.: 62 years; median: 28 years).

Five (5.9%) of the 85 people with epilepsy were initially radiologically diagnosed as “no abnormalities” ( = “MRI-negative”). In four of these, the diagnosis was established histologically. Most FCDs (62.4%) were located in the frontal lobe. The remainder were distributed among the parietal (18.8%), temporal (5.9%) and occipital lobe (2.4%), as well as the insular region (3.5%). Six (7.1%) of the FCDs were spread over two brain lobes.

Out of 85 people with epilepsy, 61 people (71.8%) were drug-resistant according to the definition of the International League Against Epilepsy (ILAE)^31^ and 50 people (58.8%) underwent epilepsy surgery. 34 people with epilepsy (68%) were classified as FCD IIb and 16 (32%) as FCD IIa, according to the Palmini classification^6^. Regarding postoperative outcomes, five (10%) were missing follow-up data. Of the remaining 45 people, 80% were seizure-free (Engel class IA) at 12-month follow-up, and only 4.4% had no worthwhile improvement (Engel class IV).

Table 1 provides a summary overview of the demographic and clinical characteristics.

**Table 1:** Summary overview of demographic and clinical characteristics.

Healthy control persons	
**Sex**	
Female	43 (50.6%)
Male	42 (49.4%)
Age at MRI-scan (mean ± SD)	33.3 ± 11.9 years (SEM 1.3, median 28)
**People with epilepsy**	
**Sex**	
Female	35 (41.2%)
Male	50 (58.8%)
**Age at epilepsy onset**	10 ± 8.3 years (SEM 0.9, median 8)
**Age at MRI-scan (mean ± SD)**	28.9 ± 12.4 years (SEM 1.3, median 25)
**Number of drug-resistant individuals**	61
**Neuroradiological Diagnosis**	
Suspicion of FCD II	78 (91.8%)
No abnormalities	5 (5.9%)
Others	2 (2.4%)
**Epilepsy surgery**	
Performed	50 (58.8%)
Not performed	35 (41.2%)
**Age at epilepsy surgery (mean ± SD)**	29.3 ± 12.4 years (SEM 1.4, median 27)
**Histopathology (Palmini)**	
FCD IIa	16 (32%)
FCD IIb	34 (68%)
**Outcome 1 year after surgery (Engel)**	
Engel class IA	36 (80%)
Engel class IB	1 (2.2%)
Engel class II	5 (11.1%)
Engel class III	1 (2.2%)
Engel class IV	2 (4.4%)
**Location**	
Frontal	53 (62.4%)
Temporal	5 (5.9%)
Parietal	16 (18.8%)
Occipital	2 (2.4%)
Insular	3 (3.5%)
Multilobar	6 (7.1%)

The clinical characteristics of all people with epilepsy and control persons are listed in the *participants.tsv* file, accessible on OpenNeuro along with the dataset^30^.

### Imaging data

MRI was performed at the Life & Brain Center in Bonn using a 3 Tesla MRI-Scanner (Magnetom Trio, Siemens Healthineers, Erlangen, Germany). As part of the comprehensive MRI protocol, a fluid-attenuated inversion recovery (FLAIR) sequence and a T1 sequence, were recorded. Due to a scanner update in early 2014, two different acquisition protocols were used. Before the update, an eight channel headcoil was used, after the update, a 32 channel headcoil was used. T1-weighted images were acquired using MPRAGE sequences. Scanning parameter before the update (“t1_iso1”) were TR = 1570 ms, TE = 3.42 ms, TI = 800 ms, flip angle 15°, matrix 256 × 256 pixel, voxel size 1.0 mm × 1.0 mm × 1.0 mm. Parameters after the update (“t1_iso0.8”) were TR = 1660 ms, TE = 2.54 ms, TI = 850 ms, flip angle 9°, matrix 320 × 320 pixel, voxel size 0.8 mm × 0.8 mm × 0.8 mm. We have used three different FLAIR protocols also resulting from system upgrades and software updates. All protocols are based on a turbo spin echo (TSE) sequence and provide 1 mm isotropic images with T2-weighted FLAIR contrast. The protocols differ in the imaging acceleration techniques (Partial Fourier, GRAPPA, or both), the image filtering applied, and the repetition times TR (5 s, 6 s, 7 s) set. Furthermore, inversion pulse application is selective only in the most recent protocol from which the most data are available.

T2-weighted FLAIR parameters before the update (“flair_tr7”) were TR = 7000 ms, TE = 372 ms, TI = 2220 ms, flip angle 150°, matrix 256 × 256 pixel, voxel size 1.0 mm × 1.0 mm × 1.0 mm. Parameters after the update (“flair_tr5”) were TR = 5000 ms, TE = 388 ms, TI = 1800 ms, flip angle 120°, matrix 256 × 256 pixel, voxel size 1.0 mm × 1.0 mm × 1.0 mm. One person with epilepsy also received a discretely different isotropic flair sequence (“flair_t6”: TR = 6000 ms, TE = 388 ms, TI = 2100 ms, flip angle 120°, matrix 256 × 256 pixel, voxel size 1.0 mm × 1.0 mm × 1.0 mm. Sequence parameters are published for all participants.

### Imaging data of people with FCD

High-resolution 3D T1-weighted MRIs were acquired for all people with FCD. The resolution of isotropic T1 sequences differed in their voxel size (mm^3^): Of the 85 people with FCD, 43 (50.6%) received T1-weighted sequences with a voxel size of 1 mm × 1 mm × 1 mm and 42 (49.4%) received T1-weighted sequences with a voxel size of 0.8 mm × 0.8 mm × 0.8 mm. Seventy-eight people with FCD (91.8%) received an isotropic FLAIR sequence. The remaining seven persons (8.2%) received a 2D FLAIR (“flair_2D”) sequence.

### Imaging data of healthy control persons

We recorded high-resolution isotropic T1-weighted and isotropic FLAIR sequences for all 85 control persons. All control persons underwent the same isotropic FLAIR sequence. Regarding T1 sequences, as for people with FCD, T1-weighted isotropic images were performed with a voxel size of 0.8 mm × 0.8 mm × 0.8 mm (78 controls, 91.8%) as well as with a voxel size of 1 mm × 1 mm × 1 mm (seven control persons, 8.2%).

An overview of the MRI sequences performed in people with epilepsy as well as the healthy control persons is given in Table 2.

**Table 2:** Overview of the MRI sequences.

	T1		FLAIR			
	isotropic T1 sequences		isotropic FLAIR sequences			2D FLAIR
	t1_iso0.8	t1_iso1	flair_tr5	flair_tr7	flair_tr6	flair_2D
Healthy control	78 (91.8%)	7 (8.2%)	85 (100%)	0 (0%)	0 (0%)	0 (0%)
people with epilepsy	42 (49.4%)	43 (50.6%)	41 (48.2%)	36 (42.4%)	1 (1.2%)	7 (8.2%)

For the visualization of epileptogenic lesions, the FLAIR sequence is the most appropriate^32^. However, morphometric analysis of 3D T1-weighted sequences is often performed as part of the preoperative workflow to detect subtle lesions^27,33^. Hence, we publish the FLAIR and the T1 sequence of all participants. Our MRI protocol has been adjusted according to changing clinical standards, resulting in different sequences being performed across the sample (see above). Details of the acquisition parameters of each scan can be found in the associated JSON files on OpenNeuro^30^.

We provided all images in NIfTI format and structured the dataset according to the BIDS specification^34^. NIfTI format is used by the neuroimaging research community^35,36^. This data format can be processed with common neuroscientific tools such as FreeSurfer or FSL, allowing easy usability of the dataset.

### Data anonymization

First, all personal identifiable information (such as name, date of birth, date of scan) were removed from the datasets. Secondly, all published MRI sequences were processed using the pydeface software^37^. This software removes all facial characteristics (so-called “defacing”). Images were visually inspected to ensure the anonymization process was successful and the neurocranium remained unaffected. Age was further categorized into age ranges of five years at onset of epilepsy, at MRI examination and, if performed, at surgery.

### Region of interest (ROI)

Ground-truth lesion masks of the dysplastic cortical regions were manually labeled based on 3D-FLAIR-weighted sequences. This was done by the collaboration of two neurologists (F.S. and T.R.), both with many years of experience in the field of epilepsy imaging, resulting in only one lesion per subject. Each lesion mask was created by one rater and reviewed by the other one. As outlined above, the general location of the lesion was determined by two neuroradiologists. Complicated cases were discussed in a joint conference of experienced neuroradiologists and epileptologists, taking into account the results of all diagnostic modalities. The definition of the ROIs was initiated in the plane where the FCD was found to be most visible and reviewer in all three plans. In case of disagreement about the extension of the ROI, all available information (MAP, SPECT, PET, invasive EEG diagnostics, postoperative MRI) was used until both reviewers agreed on the mask. Since both raters had access to all the above-mentioned information as well as the postoperative MRI images, if applicable, it was possible to create lesion masks for MRI images that were initially classified as “MRI negative” or as “no abnormalities.”

### Data processing

Preprocessing of the MRI data included conversion of the DICOM data to NIfTI format and reorganizing the NIfTI files into the Brain Imaging Data Structure (BIDS) using Dcm2Bids (https://github.com/UNFmontreal/Dcm2Bids).

Furthermore, the anonymization of the MRI-sequences was ensured by removing voxels with facial features using the pydeface software (https://github.com/1-w/pydeface). Based on the FLAIR-weighted sequences, manual labeling of the region of interest was performed using the FMRIB Software Library (FSL) editing tool to create lesion masks^38^. Detailed information about the code for preprocessing can be found in the Code Availability section.

**Fig. 1:**
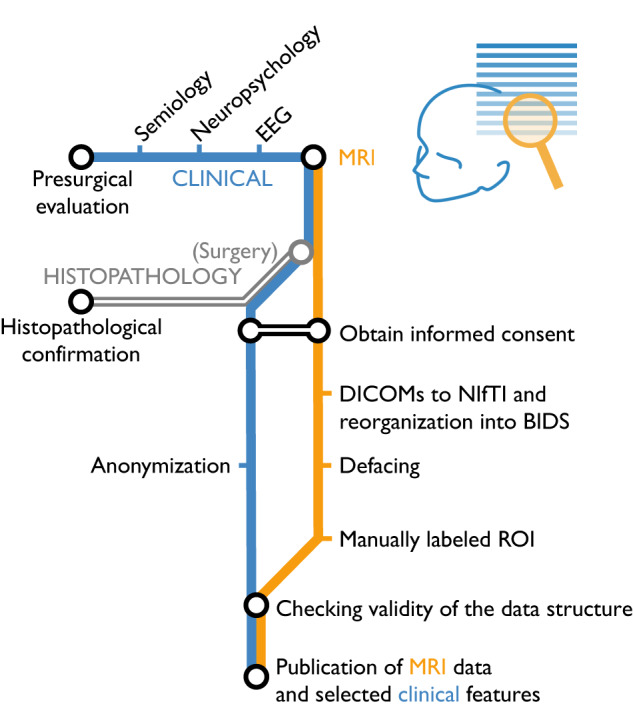
Overview of the data acquisition workflow along with data pre-processing. First, acquisition of people with FCD and control persons. Then, obtaining informed consent from all participants. Subsequently, preprocessing of MRI data and anonymization of the data.

Data collection and preprocessing workflow is shown in Fig. 1.

## Data Records

The unprocessed magnetic resonance imaging data with their associated metadata and manually labeled ROIs are publicly available on OpenNeuro^30^ (https://openneuro.org/datasets/ds004199/). The data and metadata were organized according to the machine-readable Brain Imaging Data Structure (BIDS)^34^. BIDS is an organizing and naming convention for neuroimaging data and associated metadata designed to facilitate data sharing and reuse. All neuroimaging data are available in the recommended compressed NIfTI files (.nii.gz). The tabular data file of clinical metadata is in text file format with tab-delimited values and all data descriptor files are in JavaScript Object Notation format. All MRI data files are accompanied by JSON files containing MRI acquisition parameters. Identifying metadata (e.g. name, date of birth, date of MRI scan) were removed. The top-level BIDS directory contains a *dataset_description.json* file with a description of the dataset, a descriptive *README* file, and the *participants.tsv* file (with the accompanying *participants.json* file) containing clinical and demographical metadata of subjects. The name and values of each column of the *participants.tsv* file is further described in the accompanying *participants.json* file. Missing information were indicated by “n/a” in accordance with the BIDS convention.

The imaging dataset is organized into 170 main folders. Each folder name represents an individual ID starting with “sub-” followed by a number. Each folder contains the subfolder “anat”, where the FLAIR and T1-weighted sequences in NIfTI format and the associated JSON files can be found. More detailed information on the sequences can be viewed in the associated JSON file. For people with epilepsy, the corresponding ROI in NIfTI format can also be found in the “anat” folder.

## Technical Validation

Overall, the data quality was rated as acceptable after visual inspection (by F.S.). It was first assured that the field of view comprised the complete brain, that there were no strong motion artifacts, that there were no obvious field inhomogeneities and no metal artifacts affecting the neurocranium (e.g., orthodontic retainers). The quality review was intentionally designed to resemble a visual quality review by a clinical neuroradiologist to ensure realism of the dataset. To create a benchmark dataset that was as close to reality as possible, MRI sequences that contained, e.g., slight motion artifacts were also included. In addition, the images of people with epilepsy had previously been part of the clinical routine in epileptological diagnostics and thus corresponded to a qualitative standard in everyday clinical practice. MRI data from control persons were also visually inspected and reviewed for neurological abnormalities or previously unknown diseases. After removal of the viscerocranium (i.e., “defacing”), the MRI sequences were again visually inspected to verify that the anonymization process was successful. The regions of interest were labeled and reviewed independently by two experienced raters. The dataset conforms to the Brain Imaging Data Structure (BIDS)^34^, version 1.6.0, and its validity was checked using the BIDS Validator^39^, version 1.8.9 (http://bids-standard.github.io/bids-validator/) .

**Table 3:** Code for performing the several processing steps.

Preprocessing step	Software/Tool	Software source
1. DICOM conversion into NIfTI and reorganisation into BIDS	dcm2bids	https://github.com/UNFmontreal/Dcm2Bids
2. Defacing	pydeface	https://github.com/1-w/pydeface
3. Manually labeled ROI	FSL	https://fsl.fmrib.ox.ac.uk/fsl/fslwiki

## Usage Notes

All data are publicly available and are hosted on the OpenNeuro platform (https://openneuro.org/)^30^. They are organized according to the BIDS standard^34^.

## Data Availability

Preprocessing of MRI data was performed with freely available neuroimaging tools (DICOM to NIfTI^36^ conversion and defacing^37^). The manually labeled ROIs were generated with the free software “FSL”^38^. In a final step, the correctness of the BIDS structure of our data was checked using the BIDS validator^39^. Source code and associated web link can be found in Table 3.
